# The Personalized Advantage Index: Translating Research on Prediction into Individualized Treatment Recommendations. A Demonstration

**DOI:** 10.1371/journal.pone.0083875

**Published:** 2014-01-08

**Authors:** Robert J. DeRubeis, Zachary D. Cohen, Nicholas R. Forand, Jay C. Fournier, Lois A. Gelfand, Lorenzo Lorenzo-Luaces

**Affiliations:** 1 Department of Psychology, University of Pennsylvania, Philadelphia, Pennsylvania, United States of America; 2 Department of Psychiatry, The Ohio State University, Columbus, Ohio, United States of America; 3 Department of Psychiatry, University of Pittsburgh, Pittsburgh, Pennsylvania, United States of America; Queen Elizabeth Hospital, Hong Kong

## Abstract

**Background:**

Advances in personalized medicine require the identification of variables that predict differential response to treatments as well as the development and refinement of methods to transform predictive information into actionable recommendations.

**Objective:**

To illustrate and test a new method for integrating predictive information to aid in treatment selection, using data from a randomized treatment comparison.

**Method:**

Data from a trial of antidepressant medications (N = 104) versus cognitive behavioral therapy (N = 50) for Major Depressive Disorder were used to produce predictions of post-treatment scores on the Hamilton Rating Scale for Depression (HRSD) in each of the two treatments for each of the 154 patients. The patient's own data were not used in the models that yielded these predictions. Five pre-randomization variables that predicted differential response (marital status, employment status, life events, comorbid personality disorder, and prior medication trials) were included in regression models, permitting the calculation of each patient's Personalized Advantage Index (PAI), in HRSD units.

**Results:**

For 60% of the sample a clinically meaningful advantage (PAI≥3) was predicted for one of the treatments, relative to the other. When these patients were divided into those randomly assigned to their “Optimal” treatment versus those assigned to their “Non-optimal” treatment, outcomes in the former group were superior (d = 0.58, 95% CI .17—1.01).

**Conclusions:**

This approach to treatment selection, implemented in the context of two equally effective treatments, yielded effects that, if obtained prospectively, would rival those routinely observed in comparisons of active versus control treatments.

## Introduction

The call for an increased focus on “personalized medicine [1] ” is being met by efforts across medical fields to identify predictors of treatment response [2]. In mental health, this includes recent attempts to identify genetic [3]–[6] and neuroimaging [7]–[9] indices that predict differential response to pharmacological interventions. Variables from other domains (e.g., treatment history, course, comorbidities) that predict differential response to pharmacologic versus psychological treatments have also been identified [10]–[13]. Insofar as pretreatment patient characteristics predict differential response to the interventions, patient outcomes can be optimized by the systematic use of predictive information. Published reports of prescriptive relationships tend to be limited to examinations of single pre-treatment variables or of multiple variables that are each considered in isolation. Clinicians are left with little guidance as to how to combine such predictive information, especially in cases in which the recommendations from multiple predictors conflict. As Meehl and colleagues have observed, actuarial approaches are preferred to clinical judgment in such cases [14], yet the potential for actuarial methods to inform personalized medicine by making prescriptive recommendations has not been realized.

In 1996, Barber and Muenz introduced a “matching method” to mental health researchers, with data from a randomized comparison of two different psychotherapies, cognitive behavioral therapy and interpersonal therapy. Utilizing three pre-treatment variables (marital status, avoidant personality style, and obsessive personality style), they calculated for each patient a score on a “matching factor [15].” On average, patients with positive matching scores fared better in one of the two treatments, whereas those with negative scores fared better in the other. Based on these findings, the authors recommended that clinicians consider these variables when deciding which of these treatments to recommend their patients. Their effort was a positive step towards personalizing treatment for depression, but neither their statistical approach nor the clinical recommendations it generated has been adopted by mental health researchers or practitioners.

In this paper we illustrate an approach to the use of predictive information that builds upon Barber and Muenz's efforts. The methods we describe produce point predictions of symptom severity at post-treatment for each individual in each of two interventions. The comparison of the two estimates yields an index, which we call the Personalized Advantage Index (PAI). The PAI identifies the treatment predicted to produce the better outcome for a given patient, and it provides the patient with a quantitative estimate of the magnitude by which that treatment is predicted to outperform the other. The utility of the approach is then tested by comparing the outcomes of those who had been randomly assigned to their indicated treatment versus those assigned to their non-indicated treatment.

## Methods

The approach we introduce and describe in this section of the paper can be used in any context in which patients have been randomized to two or more treatment conditions. For illustrative purposes, we use data drawn from a randomized comparative trial of cognitive behavioral therapy (CBT) versus the antidepressant medication (ADM) paroxetine in the treatment of outpatients with moderate to severe Major Depressive Disorder [16]. Each treatment was provided for 16 weeks. The trial was conducted at the University of Pennsylvania and Vanderbilt University during the period 1996 to 2002. The sampling method and outcomes have been described elsewhere [16], [17]. The data are hosted at the University of Pennsylvania. The protocol for the study, titled “Cognitive Therapy and Pharmacotherapy in Major Depression,” was approved by the respective institutional review boards at the University of Pennsylvania, Philadelphia (Protocol #034900), and Vanderbilt University, Nashville, Tennessee (Protocol #7638). The data were de-identified before use in these analyses. Following the approval of an appropriate request, the data can be anonymized and provided to researchers. Written consent was given by the patients for their information to be stored in the university database and used for research.

To simplify the presentation of our approach, we focus on data from the 154 patients for whom end-of-treatment scores were available, in either CBT (N = 50 of 60 assigned) or ADM (N = 104 of 120 assigned). End-of-treatment scores were calculated as the average of the final two scores (typically weeks 14 and 16) on the primary outcome measure, the 17-item version of the clinician-rated Hamilton Rating Scale for Depression (end-HRSD) [18]. The HRSD is the most commonly used assessment of depression symptom severity in depression treatment outcome research. In the present study, pre-treatment scores ranged from 20 to 36, where scores of 20 to 22 indicate “moderate” severity and higher scores indicate “severe” levels of depressive symptoms [19]. Differences of 3 or more points on the HRSD are considered to be “clinically significant [19].” In placebo-controlled randomized trials, medications tend to result in HRSD scores that are 2 to 3 points lower than placebo, on average, over the typical 4–8 week comparison period. This difference is associated with d-type effect size estimates of approximately 0.3 to 0.4 [20].

The end-HRSD scores in this sample were not normally distributed, which resulted in non-normal residuals when standard regression models were calculated. A square root transformation of end-HRSD resulted in distributions of raw scores and residuals that did not differ from normality, allowing the use of the standard linear regression models [21]. The values we report from the models were squared so that they would be interpretable in terms of the original HRSD scale.

### Selection of the variables to include in the models

Nine variables were found to be either prognostic or prescriptive in our sample. The details concerning these findings can be found in three published works [10]–[12]. All nine variables were measured prior to randomization. Four of these were *prognostic* [22], in that they predicted end-HRSD scores irrespective of treatment. These were: 1) pre-treatment HRSD, where higher scores predicted higher end-HRSD scores; 2) Chronic versus Non-chronic course of major depressive disorder, where chronicity was associated with poorer outcome; 3) age, where older patients fared more poorly [12]; and 4) low (<100), middle (> = 100 and <115), or high (> = 115) scores on the Shipley Institute of Living Scale, a brief measure of intellectual functioning [23], where higher scores predicted better outcomes.

The other five variables were identified as *prescriptive* in that they predicted different outcomes depending on the treatment (ADM versus CBT) that was received. These variables were detected as a statistical interaction between that variable and treatment (ADM versus CBT): 1) presence (favoring ADM) versus absence (favoring CBT) of comorbid personality disorder [11]; 2) married or cohabiting (favoring CBT) versus single; 3) employed or not expected to work versus unemployed (favoring CBT); 4) number of stressful life events (more events favoring CBT) [12]; 5) number of prior antidepressant trials, capped at 2 trials (more trials favored CBT) [10]. Like any prescriptive variable, these characteristics also produced general effects on outcome, on average across treatments [24]. The direction of these effects was as follows: being married, employed, or having a higher number of life events predicted lower end-HRSD scores, whereas having a personality disorder or having had a larger number of prior medication attempts predicted higher end-HRSD scores. Descriptive statistics for the sample as a whole and for each treatment condition separately are provided in Table 1 for each of the nine predictive variables. There were no significant differences between ADM and CBT on any of the variables (t-test for continuous variables, chi-square for categorical variables; all p's >0.1).

**Table 1:** Descriptive statistics for baseline variables.

		Total (n = 154)		ADM (n = 104)		CBT (n = 50)	
Role	Variable	Mean or %	SD	Mean or %	SD	Mean or %	SD
Prognostic	Intake-HRSD	23.8	3.2	23.8	3.2	23.7	3.4
Prognostic	Chronic Subtype	55.2%	—	58.7%	—	48.0%	—
Prognostic	Age	40.3	11.3	40.0	11.2	40.9	11.6
Prognostic	IQ						
	Lower IQ (IQ<100)	15.6%	—	19.2%	—	8.0%	—
	Mid IQ (100< = IQ<115)	52.6%	—	52.9%	—	52.0%	—
	Higher IQ (IQ> = 115)	31.8%	—	27.9%	—	40.0%	—
Prescriptive	Married	37.7%	—	39.4%	—	34.0%	—
Prescriptive	Employed	85.1%	—	86.5%	—	82.0%	—
Prescriptive	Comorbid Personality Disorder	48.1%	—	51.0%	—	42.0%	—
Prescriptive	Number Life Stressors Reported	6.6	4.8	6.7	5.1	6.3	4.3
Prescriptive	Number Prior ADM Trials^a^	0.7	0.8	0.7	0.8	0.8	0.8

ADM  =  Antidepressant Medication. CBT  =  Cognitive Behavioral Therapy. HRSD  =  Hamilton Rating Scale for Depression.

^a^ =  Capped at 2; sample breakdown for number prior medications: 0 = 52% (55% in ADM, 46% in CBT), 1 = 24% (21% in ADM, 30% in CBT), 2 or more  = 24% (24% in ADM, 24% in CBT).

### Generation of the predicted end-HRSD scores

We analyzed our data in MATLAB (The Mathworks Inc., Natick, MA). Using the GLMFIT procedure, we generated a prediction of the end-HRSD score for each participant in each of the two treatments. Hereafter we will refer to the prediction of the end-HRSD score for the treatment the participant actually received as the “factual prediction.” The “counterfactual prediction” was the estimate of the participant's end-HRSD score in the treatment he or she did not receive. Both predictions were generated by the same model, in which end-HRSD was the dependent variable.

To generate these predictions, we used techniques employed in *leave-one-out cross-validation* [25], [26]. The leave-one-out procedure (also known as a *jackknife* [27]) required the creation of 154 models, each with a sample size of 153. Main effects for “Treatment” and the prognostic and prescriptive variables, as well as terms representing the interactions of Treatment and the prescriptive variables, served as independent variables. For each of the 154 patients, the factual prediction was calculated by entering the patient's observed values on all of the independent variables into the prediction model. All values were centered using Kraemer et al. 's recommendations [24], whereby continuous measures were mean-centered, and dummy code values for dichotomous variables, including Treatment, were set at ½ and -½. We then computed each patient's counterfactual prediction by substituting the value of the other treatment (either ½ or -½ depending on the patient's actual assignment) in the Treatment main effect term, as well as in all the terms representing the interactions of Treatment and the prescriptive variables. Because each model is estimated absent any information about the patient whose scores are to be predicted, the predictions are considered to contain little or no bias [25]. In essence, the accuracy of the set of predictions is what would be expected if the procedure had been used to predict outcomes in another set of patients who were drawn randomly from the same population of patients, assuming they would be assigned to the same treatments in the same way (i.e., randomly) [27].

### Properties of the predictions that will be examined

Using the predicted scores, we estimated: (1) the “true error” of the factual predictions (i.e., the mean of the absolute value of the difference between the observed scores and factual predictions); (2) the standard error of the set of predictions; and (3) the magnitude of the predicted difference, for each patient, of receiving the treatment with the greater predicted benefit (Optimal) versus the other (Non-optimal) treatment. This last value is an index of “predicted advantage” which we call the Personalized Advantage Index (PAI). Because each individual is left out of the model from which their end point values are predicted, and because the Optimal treatment predicted for an individual is not tied to the treatment actually received, we can take advantage of the initial randomization of patients to treatments in order to test the utility of the PAI by comparing the mean observed difference, in end-HRSD units, between the set of patients who had been randomly assigned to their Optimal treatment versus those who had been assigned to their Non-Optimal treatment.

### A worked example of the approach

Tables 2, 3, 4 illustrate how the procedure generated the predictions for CBT and ADM, using one of the 154 patients from the sample. This patient was selected because the PAI, the observed end-HRSD, and the prediction error were near the mean for the sample. Table 2 shows how this patient's values on two of the four prognostic variables (low intake HRSD; high intellectual level) predicted better outcome (i.e., lower end-HRSD scores, as indicated by negative values of a*b), whereas values on the other two prognostic variables (older; chronic course) predicted poorer outcome for this patient. As can be seen in the lower portion of Table 2, the patient's values on three of the prescriptive variables (unmarried, unemployed, two prior ADM trials) predicted poorer outcome irrespective of treatment. On two others (three life stressors, no comorbid Personality Disorder), the values of a*b are close to zero, indicating little influence on their own in the prediction of outcome.

**Table 2:** How the weights associated with prognostic and prescriptive variables combine with a patient's values to contribute to the calculation of the patient's Personalized Advantage Index.

Variable	Patient's value	Transformation	Input value (a)	Beta in LOO model (b)	a*b
Intercept	n/a	n/a	1	3.15	3.15
Intake-HRSD (M = 23.8)^a^	20	Mean-centered	−3.75	0.05	−0.20
Age (M = 40.3)^a^	56	Mean-centered	15.67	0.01	0.20
IQ (Low, Middle, High)^a^	High	−1,0,1	1	−0.18	−0.18
Chronic Subtype^a^	Yes	−.5, .5	0.5	0.39	0.19
Marital Status^b^	Unmarried	−.5, .5	−0.5	−0.45	0.22
Employment Status^b^	Unemployed	−.5, .5	−0.5	−0.50	0.25
Number Life Stressors Reported (M = 6.57)^b^	3	Mean-centered	−0.65	−0.07	0.04
Comorbid Personality Disorder^b^	No	−.5, .5	−0.5	0.17	−0.08
Number Prior ADM Trials (capped at 2; M = 0.72)^b^	2	Mean-centered	1.28	0.28	0.35
Total, for use in end-HRSD predictions^c^				Sum a*b	3.96

LOO  =  Leave One Out. ADM  =  Antidepressant Medication. HRSD  =  Hamilton Rating Scale for Depression.

a =  Prognostic variable.

b =  Prescriptive variable.

c =  See Table 4.

**Table 3:** The treatment (Tx) main effect and interactions of Tx with the prescriptive variables.

		Tx = CBT		Tx = ADM	
Variable	Beta in LOO model (b)	Input (c)	b*c	Input (m)	b*m
CBT (0.5) or ADM (−0.5)	−0.42	0.5	−0.21	−0.5	0.21
Tx*Marital Status	−1.10	−0.25	0.27	0.25	−0.27
Tx*Employment Status	1.03	−0.25	−0.26	0.25	0.26
Tx*Life Stressors	−0.35	−0.32	0.11	0.32	−0.11
Tx*Personality Disorder	0.66	−0.25	−0.16	0.25	0.16
Tx*Prior ADMs	−0.17	0.64	−0.11	−0.64	0.11
Total, for use in end-HRSD predictions^a^		Sum b*c	−0.35	Sum b*m	0.35

LOO  =  Leave One Out. CBT  =  Cognitive Behavioral Therapy. ADM  =  Antidepressant Medication. Tx  =  Treatment. HRSD  =  Hamilton Rating Scale for Depression.

a =  See Table 4.

**Table 4:** How the estimates from Tables 2 and 3 combine to produce a patient's estimated end-HRSD scores in CBT and ADM, respectively, and the PAI.

Tx = CBT	Value	Source	Value	Tx = ADM
3.96	<—Sum a*b	Table 2	Sum a*b—>	3.96
−0.35	<—Sum b*c	Table 3	Sum b*m—>	0.35
3.6	Sum of sums			4.3
13.0	Predicted end-HRSD^a^			18.6
	PAI = 5.6, favoring CBT			

Tx  =  Treatment. CBT  =  Cognitive Behavioral Therapy. ADM  =  Antidepressant Medication. HRSD  =  Hamilton Rating Scale for Depression. PAI  =  Personalized Advantage Index; the difference between the predictions for CBT and ADM, in end-HRSD units.

a =  The square of the model output.

Table 3 shows how treatment affects the prediction of outcome, both as a main effect and in interactions with each of the five prescriptive variables. This patient's values on three of the five prescriptive variables indicated CBT as the Optimal Treatment (unemployed, no comorbid Personality Disorder, two prior ADM trials) as reflected in the negative b*c values. Values on the other two variables indicated ADM as the Optimal Treatment (unmarried, three life stressors), reflected in negative b*m values. The model's outputs (see Table 4) indicate that the patient's predicted end-HRSD is 13.0 in CBT and 18.6 in ADM. The Personalized Advantage Index (PAI) for this individual is 5.6 in favor of CBT; it represents the difference between the endpoint scores predicted for each treatment.

## Results

**Figure 1:**
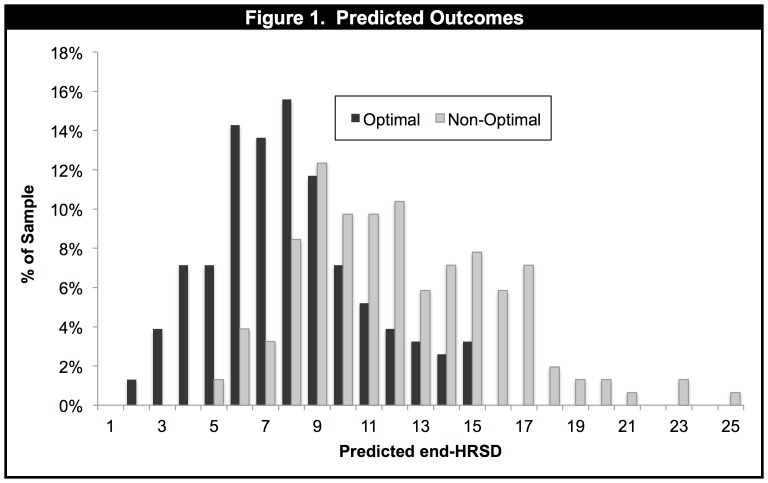
Frequency histogram showing predicted end-HRSD scores for each patient in their Optimal and their Non-Optimal treatment, as indicated by the treatment selection algorithm.

The true error of the end-HRSD score predictions (the average absolute difference between the predicted and actual scores, across the 154 patients) was 4.9. The standard error of prediction was 6.2. Figure 1 displays the distributions of the predicted end-HRSD scores for the Optimal and Non-Optimal treatments across the 154 patients.

**Figure 2:**
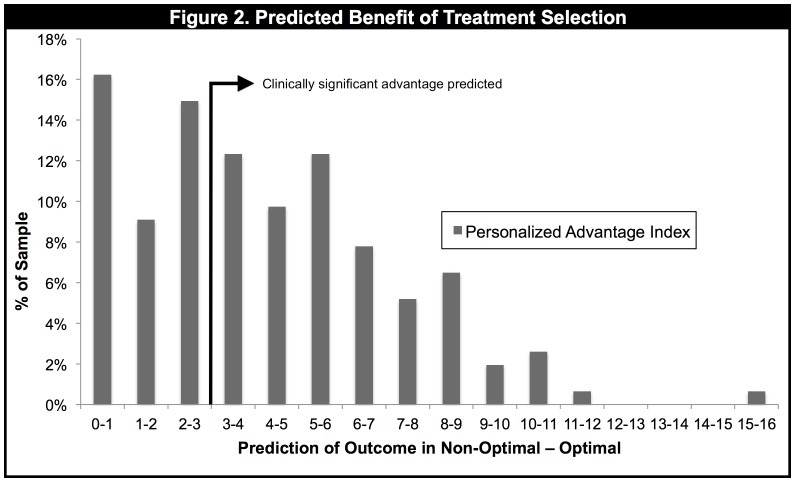
Frequency histogram showing Personalized Advantage Index (PAI) scores for all patients in the sample.

The distribution of PAI scores is shown in Figure 2. The average PAI was 4.2 (SD = 2.9), representing a 4.2 point difference in end-HRSD scores between the Optimal treatment (predicted mean = 7.4, SD = 3.0) versus the Non-Optimal treatment (predicted mean = 11.6; SD = 3.9). Note that a patient's PAI can be as low as 0, which would occur if the same outcome is predicted for both treatments, irrespective of whether high or low end-HRSD scores are predicted. As can be seen, whereas for some patients the predicted advantage of being assigned to their Optimal treatment was large, for others it was very small. For 62 (40%) of the patients, the PAI did not meet the National Institute for Health and Care Excellence (NICE) criterion (three points on the HRSD) for a “clinically significant” difference. For such patients, little weight would be given to the model's predictions in a treatment selection decision; other factors (e.g., cost or patient preference) would likely be used to guide treatment. We test our approach, therefore, using the full sample of 154 patients as well as a reduced sample of those 92 patients (60%) whose PAI was “clinically significant.”

**Figure 3:**
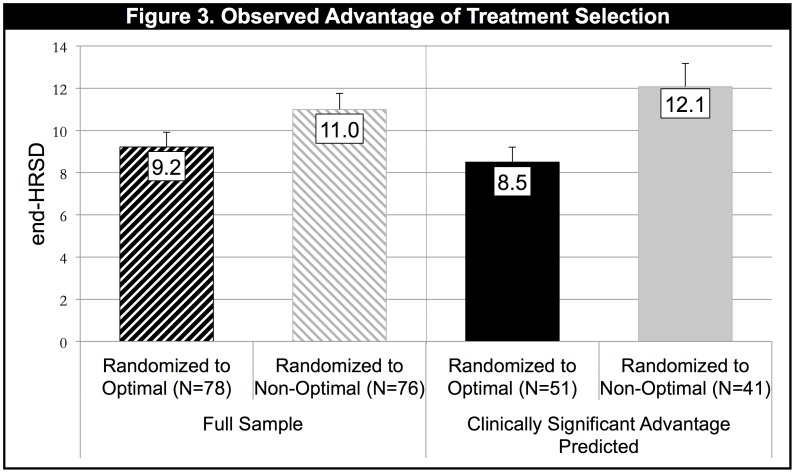
Comparison of mean end-HRSD scores for patients randomly assigned to their Optimal treatment versus those assigned to their Non-Optimal treatment. The left side gives the results for the full sample. The right side includes only patients for whom the algorithm predicted a clinically significant advantage on the PAI of ≥3.

The left side of Figure 3 shows, for the full sample, a comparison of the average end-HRSD score for those assigned randomly to their Optimal treatment versus those assigned to their Non-Optimal treatment. Given that in 40% of the sample the Optimal versus Non-Optimal difference was quite small, it is not surprising that the observed difference between the Optimal and Non-Optimal means in the full sample was relatively small; they differed at the level of a nonsignificant trend (mean difference  = 1.78; pooled SD  = 6.38; t = 1.73, 152, p = .09; d = .28, 95% confidence interval −.04 to .60). The right side of the figure gives the means for the 60% of the sample for whom the predicted advantage of the Optimal treatment was clinically significant. Here, the observed mean difference was both clinically and statistically significant (mean difference  = 3.58; pooled SD  = 6.12; t = 2.84, 90, p = .006; d = .58, 95% confidence interval .17 to 1.01).

## Discussion

The method we have illustrated can be used to optimize treatment selection in any context in which: a) more than one intervention is under consideration, b) comparative outcome data are available, and c) pre-treatment factors can be identified that predict outcomes differentially across the interventions. In our example, a randomized comparison of cognitive behavioral therapy versus medications for depression, the treatments produced similar average levels of symptom reduction [16]. We used our approach to predict, for each patient, which treatment was more likely to lead to a better outcome. We then examined the results of the natural experiment that occurred whereby some patients had been randomized to their Optimal treatment and some to their Non-optimal treatment. In line with our hypothesis, patients randomized to their Optimal treatment tended to fare better than those who were randomized to their Non-optimal treatment.

When we restricted our test of the method to those for whom the PAI was clinically significant, the advantage of assignment to the Optimal treatment was, in effect size terms, approximately twice the difference reported in a recent systematic review of antidepressant drug versus placebo comparisons [28], and larger than the average effect size observed between control and active treatments utilized in general medical contexts [29]. This result exemplifies an important feature of the approach: the ability to identify individuals for whom the difference in outcome between treatments is likely to be large, as well as those for whom the predictions are similar and, thus, should not be given substantial weight in a choice between the two treatments. In applications of this approach, other factors, such as patient preference or treatment costs would likely weigh heavily in treatment selection decisions, when the PAI is small. It is important to emphasize that both ADM and CBT are evidence-based treatments for depression. Thus, all patients, including those identified as having received what for them was their Non-optimal treatment, received what is considered, absent any contraindications, a valid and appropriate treatment.

Although we could not conduct a prospective test with our data, we approximated a critical feature of such a test by leaving each patient's data out of the model that was used to make predictions for him or her. Thus, the benefits of treatment optimization we observed should provide a good estimate of the advantage that would have accrued to future patients from the same population had the prediction algorithm been used to assign them to the same treatments we studied. In a real world clinic, a consecutive series of patients would be randomized to one of two evidence-based treatments. Patient outcomes would be tracked, and baseline characteristics would be used to generate the predictive algorithm that would inform treatment decisions for future patients. The weight given to each new patient's treatment recommendation would depend on the magnitude of the PAI generated by the algorithm.

A true prospective test of our approach would begin with a randomized trial of two interventions. A predictive model, as we have described here, would be derived from the data obtained during the randomized trial. The model would then be tested in sample of patients who seek treatment in the same clinic in which the randomized trial was performed, using the same treatments. Outcomes of patients who are randomized to one of two conditions would then be compared: (a) those whose treatment is determined by random assignment, as in the first phase of the study; versus (b) those whose assignment is determined by the output of the predictive algorithm that was generated in the first phase.

It is often challenging to identify prescriptive variables that will replicate in a different population. Several features of the study from which the present data were drawn likely contributed to the strong prescriptive findings we obtained, and might also support a successful effort to replicate them. Cognitive behavioral therapy and antidepressant medications are both effective interventions for depression, but they are very different methods of treatment that likely work through different mechanisms [30]. We therefore expected to be able to identify prescriptive variables, especially given that several of the pre-treatment variables were included in the intake battery precisely because prior research had suggested that they predict differential response to these treatments. In comparisons of two treatments that work through similar mechanisms, such as might be true of two medications that operate on similar neurotransmitter systems, the power of this approach, or any approach that is contingent on the presence of significant treatment-by-patient-characteristic interaction effects, would likely be limited.

The variables in our example comprised information from structured interviews, self-report questionnaires, and demographic forms, any of which can readily be obtained in a routine clinical setting. Other groups have begun to explore the potential of genetics or neuroimaging to inform treatment decisions in depressed patient populations [5], [31]–[33]. In pharmacogenetic and pharmacogenomic studies, perhaps because the interventions included are mechanistically similar, the effects have thus far been small [6]. In principle, however, information from multiple different kinds of measures could be combined using the procedures we describe above in order to provide more accurate predictions than could be generated from any one predictor considered in isolation.

The potential for neuroimaging-based treatment selection was evidenced recently in an investigation by Mayberg and colleagues, who explored the associations between pre-treatment brain activation and outcomes in a randomized comparison of CBT and ADM [32]. They reported that indexes of brain activity in six regions, as assessed with positron emission tomography, were associated with differential response to the two treatments. They focused on their strongest finding, which was obtained from the right anterior insula. Patients who remitted with CBT, as well as those who did not remit with ADM, exhibited relatively low activity in this region, whereas those who remitted in ADM, as well as those who did not remit in CBT, exhibited relatively high activity in that area. These findings represent a major contribution to prediction of treatment response. However, they examined each of the six indexes in isolation, and thus did not make maximal use of the predictive information provided from the multiple brain regions. Moreover, their approach does not allow for the quantification of benefit from treatment matching. As we have shown, some patients would be expected to derive comparable benefits from either treatment, whereas for others there would be little if any difference in outcomes expected between the two treatments. Considering these factors, it is not clear how their findings, or any set of findings in which multiple different predictors are identified, would be used in clinical decision-making on their own. Conversely, our approach produces a clinically interpretable index of the size of the expected difference in outcomes between the treatments. Future studies of neuroimaging or genetic markers as differential predictors of treatment response would do well to include a wide variety of variables and modalities in pre-treatment assessments and to take advantage of the multivariate nature of the set of potential predictors [34]–[37].

Biostatisticians have described analytic frameworks to identify prescriptive (moderator) variables [38], [39], but less attention has been paid to the development of procedures to translate prescriptive findings into clear, actionable recommendations for individual patients. We were alerted to the points of contact between our approach and that of Barber and Muenz [15] while we were developing and testing our method, at which time a thorough review of the literature revealed no further developments along these lines in the mental health field. Only after an extensive review of the literature in other medical fields did we locate similar efforts, in oncological medicine [40]–[42]. To our knowledge, none of that prior work has been developed further or applied to the differential prediction of individual patient outcomes.

The time is right for the revival, further development, and application of these methods, first introduced 35 years ago [40], as such approaches are suited perfectly to advance the goals of personalized medicine. With the present effort we hope to inspire renewed interest across medical fields in the development and application of prescriptive algorithms that combine multiple sources of information to yield estimates of patients' outcomes in more than one treatment. This approach promises to enhance therapeutics by promoting the selection of the best treatment among available options, with the additional feature that it provides quantitative estimates of the benefits that can be expected when such an algorithm is implemented.
